# Influence of Homogenization on Phase Transformations during Isothermal Aging of Inconel 718 Superalloys Fabricated by Additive Manufacturing and Suction Casting

**DOI:** 10.3390/ma16144968

**Published:** 2023-07-12

**Authors:** Yunhao Zhao, Wei Xiong

**Affiliations:** Physical Metallurgy and Materials Design Laboratory, Department of Mechanical Engineering and Materials Science, University of Pittsburgh, Pittsburgh, PA 15261, USA; yunhao.zhao@pitt.edu

**Keywords:** intermetallic, laser processing, kinetics, phase transitions, superalloy

## Abstract

The attainment of the desired strength of the Inconel 718 superalloy heavily relies on the isothermal aging process, which plays a critical role in achieving the anticipated hardening effect. Surprisingly, there remains a dearth of dedicated studies investigating the influence of homogenization on phase transformations during the isothermal aging process, leaving a gap in the knowledge required to guide the design of post-heat treatment strategies. Addressing this gap, our work investigates the impact of homogenization time on phase transformations during isothermal aging at 730 °C in Inconel 718 alloys produced via additive manufacturing (AM) and suction casting (SC) methods. Intriguingly, we observe contrasting behaviors in the particle size of γ″ and γ′ in aged samples, depending on the homogenization time and the alloy processing method. Specifically, in AM alloys, extended homogenization time leads to an increase in the particle size of γ″ and γ′, whereas the opposite trend is observed in SC alloys. Furthermore, despite undergoing the same heat treatment, the AM alloys exhibit smaller particle sizes but higher precipitate number densities compared to the SC alloys, resulting in superior hardness. Notably, pronounced grain refinement during aging is evident in 1 h homogenized SC samples under 1180 °C, warranting further investigations into the underlying mechanisms. This study elucidates the crucial role of homogenization in attaining the desired microstructure following subsequent aging processes. Moreover, it offers novel insights for developing post-heat treatment strategies for superalloys.

## 1. Introduction

The Inconel 718 superalloy has a broad application in aerospace due to its exceptional mechanical properties and corrosion resistance during high-temperature service. Recently, a rapid growth in research on the Inconel 718 alloys fabricated by additive manufacturing (AM) has occurred [1]. The AM approach builds components layer-by-layer to achieve sophisticated geometric features, and the waste of feedstocks during the AM process can be notably reduced compared with traditional subtractive manufacturing [2]. Moreover, the high printability of Inconel 718 makes it particularly suited to being produced by the AM approach [1,3,4]. However, the Inconel 718 builds produced by AM cannot be strengthened through work hardening, which highlights the importance of the post-heat treatment optimization for the microstructure engineering of these alloys.

As Zhao et al., summarized [5], the heat treatments for conventional wrought/cast Inconel 718 alloys usually contain one homogenization (solutionization) process at 970~1065 °C, and two subsequent aging steps. The primary purpose of the homogenization is to dissolve the detrimental Laves_C14 phase ((Ni, Fe, Cr)_2_(Nb, Ti, Mo), hexagonal), which is formed during the solidification process due to the Nb segregation along the grain boundaries, and to release the Nb into the γ (fcc_A1) matrix to facilitate the precipitation of the strengthening phases. During homogenization, NbC carbides (fcc_B1) usually coexist with the γ matrix, as they are stable at elevated temperatures [5]. The first and second aging steps are generally performed at 718 and 621 °C, respectively [5,6]. The primary precipitates during the aging steps are the plate-shaped γ″ phase (Ni_3_Nb, bct_D0_22_), which acts as the principal strengthening phase, and the sphere-shaped γ′ (Ni_3_(Al,Ti,Nb), fcc_L1_2_) phase, which is the minor strengthening phase [7,8]. Meanwhile, the needle-shaped δ phase (Ni_3_Nb, D0_a_) usually forms during aging along grain boundaries, whereas these precipitates are detrimental to the mechanical properties [9,10].

In the AM Inconel 718 alloys, homogenization performed at a higher temperature, e.g., 1180 °C [5,11], than traditional (~1060 °C) has been found to effectively dissolve the Laves_C14 phase and remove the columnar grain texture by triggering the recrystallization process; thus, high-temperature homogenization has become a promising optimal heat treatment to improve the material properties of AM alloys [5,11,12,13,14,15,16,17,18,19]. However, considering the fact that different homogenization conditions can lead to various initial microstructures in an alloy before the aging process, they may further affect the microstructure and properties of the aged alloy. Some research has been conducted to understand these relationships in the AM Inconel 718 alloys. Tucho and Hansen [16] investigated the effects of different homogenization temperature (970~1250 °C) and time (1 and 7 h at each temperature) combinations on the microstructure after traditional two-step aging in SLM-fabricated (selective laser melting) Inconel 718. They found that an increase in homogenization time at a particular temperature could cause the coarsening of γ″ and degrade the hardness of the materials. Huang et al. [17] performed homogenizations at 980~1280 °C for 1 h on the SLM Inconel 718 before traditional aging, and developed a relationship ($t=\frac{1}{3266}exp\frac{21642}{T}$) between the minimum temperature T and time t for Laves phase dissolution. They also found that the dissolution of the Laves phase can improve the ductility and accelerate phase transformations during the aging process. Raghavan et al. [19] studied the homogenization temperature (at 1040, 1100, and 1200 °C, with homogenization time not indicated) influence on the aged SLM Inconel 718 alloys, and found that an increased homogenization temperature can reduce the strength but can increase the ductility of the aged alloys. Specifically, the strength of the aged alloy homogenized at 1200 °C dropped significantly, but its ductility increased by 20% compared to that homogenized at 1040 °C. Ni et al. [15] concluded that high-temperature homogenization at 1100 °C for 1 h could increase the isotropy of the microstructure and mechanical properties of the SLM Inconel 718 alloys. Sui et al. [18] focused on the effect of different homogenization time (at 1100 °C with 0.5 and 1 h) on the traditionally aged Inconel 718 alloys processed by laser metal deposition. The longer homogenization time was found to increase the recrystallization degree and to form twins, which were not observed in the sample under short-term homogenization. The mechanical properties were comparable.

Although the studies mentioned above have initiated the research on the homogenization influence on aged AM Inconel 718 alloys, they primarily focus on the two-step aging process following the standard heat treatment strategies. It remains unclear how the microstructure evolution proceeds during each aging step, as do the impacts of homogenization on the phase transformations thereof. The absence of such fundamental research impedes further post-heat treatment optimization for the additively manufactured Inconel 718. In this work, the homogenization-affected microstructure and property evolutions during the one-step aging process are investigated in the Inconel 718 alloys processed by laser powder bed fusion (PBF-LB/M). The effects of different homogenization durations on phase transformations, grain morphology evolution, and microhardness are investigated. Alloys made by suction casting (SC) are used to benchmark the results generated from AM alloys. The comparison between the AM and SC alloys can also be conducive to understanding the microstructure evolution due to the differences in the initial microstructure. The results demonstrate that homogenization choice and manufacturing method can influence phase transformations and mechanical properties during aging. 

## 2. Experiments

The AM Inconel 718 alloy was printed, following the PBF-LB/M method, using an EOS M290 DMLS system (Direct Metal Laser Solidification, EOS GmbH, Krailling, Germany) with default parameters specifically optimized for Inconel 718. The Inconel 718 powder is virgin powder manufactured by Praxair Surface Technologies, Inc. (Indianapolis, IN, USA). The d_10_, d_50_, and d_90_ particle sizes of the powder are 18.5, 20, and 44 µm, respectively. The laser power is 285 W, the scan velocity is 960 mm/s, and the hatching space is 0.11 mm, with the hatching lines between adjacent layers having a rotation angle of 67°. More details of printing parameters are given in the Supplementary Materials. The suction-cast alloys were prepared using an arc-melter (ABJ-338, Materials Research Furnace Inc., Allenstown, NH, USA) under an argon atmosphere. The nominal compositions of the AM and SC alloys are listed in Table 1, from which it can be observed that the compositions of the two alloys are relatively close. Both alloys were sectioned into cuboid shapes via wire cutting using an EDM (electrical discharge machining, Mitsubishi Group, Chiyoda City, Tokyo, Japan) machine, and were encapsulated into quartz tubes with a backfilled Ar protective atmosphere for heat treatment. The suction-cast alloy samples were cut off from rod suction-cast Inconel 718 materials with a length of 40 mm and a diameter of 11 mm. The AM alloy samples were cut off from a build with dimensions of 100 mm (length) × 10 mm (width) × 45 mm (height).

**Figure 1:**
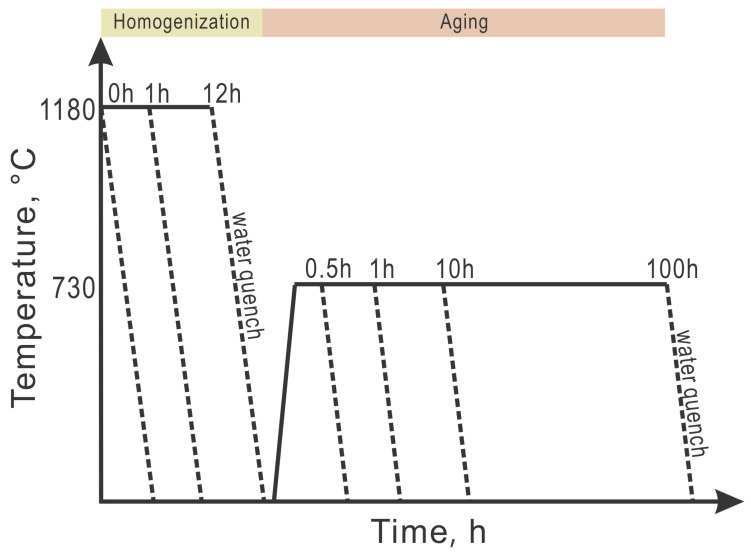
Temperature diagram of heat treatments in the present work.

In order to investigate the effects of homogenization time on the aging process, the AM alloys were divided into three groups. The first group reserves the as-printed microstructure without homogenization, and the samples were aged at 730 °C for 0.5, 1, 10, and 100 h, followed by water quenching. These samples are labeled as AM0-05, AM0-1, AM0-10, and AM0-100, as shown in Table 2. Similarly, the suction-cast samples under the same heat treatment conditions were prepared, and are noted as SC0-05, SC0-1, SC0-10, and SC0-100 in Table 2. The second group was subjected to homogenization for 1 h at 1180 °C, followed by water quenching before the same aging conditions as the first group, and are thus labeled AM1-05, AM1-1, AM1-10, and AM1-100 for the alloys produced by PBF-LB/M, and SC1-05, SC1-1, SC1-10, and SC1-100 for suction-cast alloys, as shown in Table 2. The third group of alloys prepared by AM and SC methods, experiencing the longest homogenization of 12 h, is listed in the same table. A plot of temperature vs. time summarizing the heat treatment options is given in Figure 1.

The heat-treated samples were then prepared by the metallographic procedures required for microstructure characterization, which included a grinding process using sandpaper with 400, 600, 800, and 1200 grit, and a polishing process with 1 µm and 0.05 µm as final steps. The microstructure on the longitudinal planes, i.e., the XZ plane (Z indicates the build direction), was studied for the AM alloys. SEM (scanning electron microscopy) was conducted on a Zeiss Sigma 500 VP electron microscope (Carl Zeizz AG, Oberkochen, Baden-Württemberg, Germany), and the EBSD (electron backscatter diffraction) mapping was obtained from an FEI Scios Dual-Beam system (FEI Company, Hillsboro, OR, USA). The EBSD mapping area was 800 × 800 μm with a step size of 1.6 μm to determine the grain morphology. To quantitatively investigate the precipitation behaviors of the nanosized γ″ and γ′ phases, the aged samples were etched using a solution of 50 mL C_3_H_6_O_3_ + 30 mL HNO_3_ + 2 mL HF [20] that can react with the Nb-rich phases; the phases were then observed using SEM. The particle morphology information of γ″ and γ′ phases was analyzed using the Image-J software 1.53a package. Vickers hardness testing was performed using a Leco LM-800 tester (Leco Corporation, St. Joseph, MI, USA) under a 50 or 100 gf load for 10 s. Hardness was found not to change along the Z axis in the as-printed build. For each sample with a specific heat treatment condition, nine indents were performed on the XZ plane in a 3 × 3 hardness map with an adjacent indent distance of 60 µm. The average value was taken as the hardness reading of the sample thereof.

## 3. Results and Discussion

### 3.1. Phase Transformations in the AM Alloys

**Figure 2:**
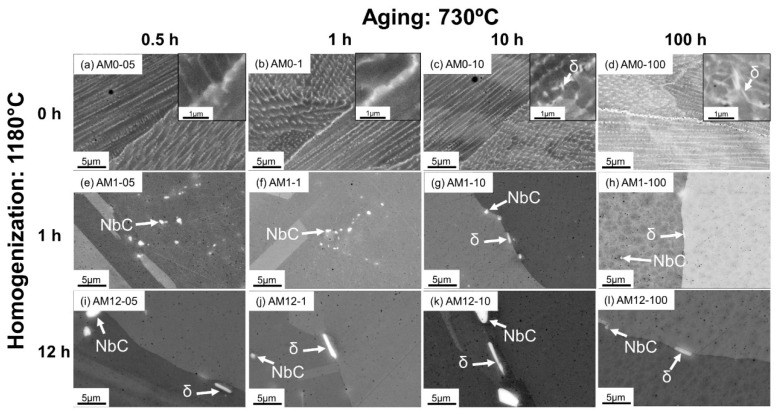
SEM-BSE micrographs of the direct-aged AM samples ((**a**) AM0-05; (**b**) AM0-1; (**c**) AM0-10; (**d**) AM0-100), the aged AM samples homogenized at 1180 °C for 1 h ((**e**) AM1-05; (**f**) AM1-1; (**g**) AM1-10; (**h**) AM1-100), and the aged AM samples homogenized at 1180 °C for 12 h ((**i**) AM12-05; (**j**) AM12-1; (**k**) AM12-10; (**l**) AM12-100).

**Figure 3:**
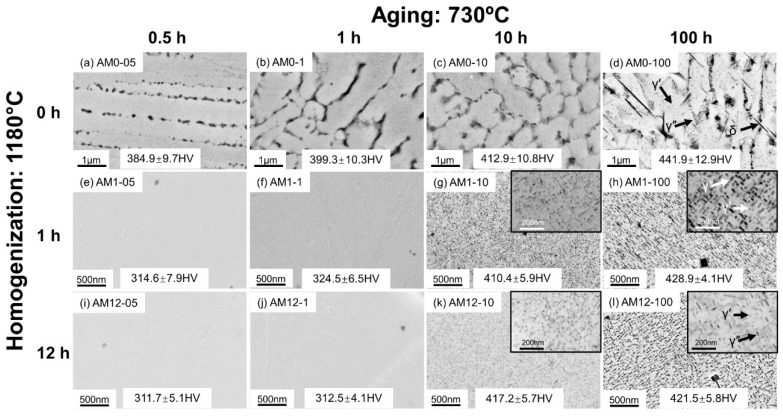
SEM-SE micrographs of the direct-aged AM samples after etching ((**a**) AM0-05; (**b**) AM0-1; (**c**) AM0-10; (**d**) AM0-100), the aged AM samples homogenized at 1180 °C for 1 h after etching ((**e**) AM1-05; (**f**) AM1-1; (**g**) AM1-10; (**h**) AM1-100), and the aged AM samples homogenized at 1180 °C for 12 h after etching ((**i**) AM12-05; (**j**) AM12-1; (**k**) AM12-10; (**l**) AM12-100).

The phase transformations in the AM alloys were characterized by SEM, and their microstructures are shown in Figure 2 and Figure 3. Figure 2 presents the backscattered electron (BSE) images from SEM on AM samples under different post-heat treatment conditions. The microstructure analysis was directly performed on the as-polished sample surface. Figure 3 shows the SEM secondary electron (SE) images taken from the same samples shown in Figure 2 after chemical etching to unveil strengthening phases γ″ and γ′. It should be pointed out that TEM (transmission electron microscopy) is widely used to characterize phase attributes for nanoscale γ″ and γ′ precipitates. For instance, Papadaki et al. [21] used in situ TEM observation to investigate the Gibbs–Thomas effect on nucleation, growth, and coarsening process of γ′ in a Ni-based superalloy, and the particle attributes of primary, secondary, and tertiary γ′ were measured. Theska et al. [22] used high-resolution TEM technology to investigate precipitation behavior of Inconel 718 at early stages of direct aging. However, although TEM is able to gain more in-depth information for the nanoscale precipitates, the intricate sample preparation and operation technology lower the research efficiency when microstructure characterization is needed for a large number of samples. As an alternative, SEM technology has been proven to be effective for γ″ and γ′ characterization in Inconel 718, with a relatively higher efficiency in regards to precipitation morphology investigation [23], which is capable of substantiating the discussions and conclusions of the present work. Therefore, SEM was used in this work to achieve accurate measurements while ensuring a statistically significant number of particles for a representative analysis.

As shown in Figure 2a–d, in the samples aged at 730 °C without homogenization (i.e., the direct-aged samples), the microstructure contains very fine columnar and cellular subgrains inside the relatively larger grains. Bright Laves_C14 precipitates with an irregular shape can be observed to form along the subgrain boundaries as a result of the Nb segregation during the solidification process. The microstructure in the direct-aged samples is identical to that in the as-built samples, as reported in [5,17,24], indicating that direct aging at a moderate temperature cannot completely remove the as-printed dendritic structures. However, after aging for 10 h, the needle-shaped δ particles precipitate in the region rich in the Laves_C14 phase, as indicated by the inset of Figure 2c. Such particles further grow after long-term aging for 100 h, as shown in the inset of Figure 2d. The Laves_C14 phase is unstable in Inconel 718, and will dissolve into the γ matrix during the isothermal heat treatment. The dissolution of the Laves_C14 phase releases the Nb into γ, which promotes the precipitation of the stable δ phase at 730 °C. Meanwhile, the δ precipitation is further promoted by a large number of grain boundaries inherited from the as-printed states, since grain boundaries are the preferred nucleation sites for the δ phase.

Evidently, as shown in Figure 2e–l, after homogenization, the microstructure in the aged samples notably deviates from that in the direct-aged samples. With 1 h homogenization, the Laves_C14 phase can be eliminated, leaving the stable NbC carbides in the γ matrix. The δ precipitates were found to form obviously after 10 and 100 h aging (Figure 2g,h), with the particle size being larger than their counterparts in the direct-aged samples (Figure 2c,d). However, the number density of the δ particles can be observed to be much reduced, which is attributed to the reduction in the grain boundary fraction after homogenization. After 12 h homogenization, the precipitation of the δ during aging is found to evidently start with 0.5 h aging, as shown in Figure 2i, which is faster than that in the other homogenization conditions. The number densities of the δ particles are still low in the aged samples; thus, the δ phase is not supposed to cause an impact on the material properties under this circumstance. The experimental observation indicates the requirement of homogenization optimization, which can help eliminate the Laves_C14 phase and inhibit δ phase precipitation during the aging process, as both phases are detrimental to the mechanical performance.

The precipitation behaviors of the nanosized γ″ and γ′ phases are analyzed by microstructure characterization in the etched samples, as shown in Figure 3. The light-black dots in the matrix of Figure 3a indicate the trace of γ″/γ′ small precipitates in sample AM0-05. As a result, the hardness of this sample is 384.9 ± 9.7 HV, which is about 50 HV higher than that of 338.2 ± 9.7 HV in the as-built sample, in which no γ″/γ′ precipitates can be observed. As the aging proceeds, the γ″/γ′ phases keep precipitating and grow sufficiently in sample AM0-100 after 100 h; meanwhile, the hardness value is increased to 441.9 ± 12.9 HV, demonstrating the precipitation hardening caused by γ″/γ′. As seen in the inset of Figure 3d, the plate-shaped γ″ particles tend to form near the grain boundaries, and are close to the larger needle-shaped δ particles, as the Nb content in such regions is relatively higher because of the dissolution of the Laves_C14 phase. In contrast, due to the segregation of Nb at the subgrain boundaries, the relative content of Al and Ti, which are critical components of the γ′ phase, increases in the interior of the subgrains, resulting in the sphere-shaped γ′ particles primarily precipitating inside such areas, as illustrated in Figure 3d. The different precipitation behaviors of γ″ and γ′ imply that the heterogeneous elemental distribution in the as-built sample leads to the inhomogeneous precipitation of the strengthening phases.

Compared with the direct-aged samples, Figure 3e–l shows that homogenization can significantly impact precipitation kinetics during aging. For the aged samples homogenized after 1 h at 1180 °C, the γ″/γ′ phases are absent after aging for 0.5 (sample AM1-05) and 1 h (sample AM1-1) at 730 °C, as shown in Figure 3e,f, respectively. A significant increase in the number density of strengthening precipitates can be observed in sample AM1-10 with 10 h aging at 730 °C (Figure 3g). In contrast, the precipitates distribute evenly inside the γ matrix, but have not grown sufficiently, and it is hard to differentiate the particles in this sample. After 100 h aging at 730 °C (Figure 3h), the γ″ and γ′ phases have grown large enough to be differentiated with γ″ as the plate shape and γ′ as the spherical shape. The aged samples after 12 h homogenization (Figure 3i–l) have very similar precipitation behaviors to the corresponding aged samples with 1 h homogenization. However, the precipitation attributes, i.e., the particle size and number density in the aged samples, are found to be impacted by the homogenization time, which will be discussed in detail in Section 3.3.

### 3.2. Phase Transformations in the SC Alloys

**Figure 4:**
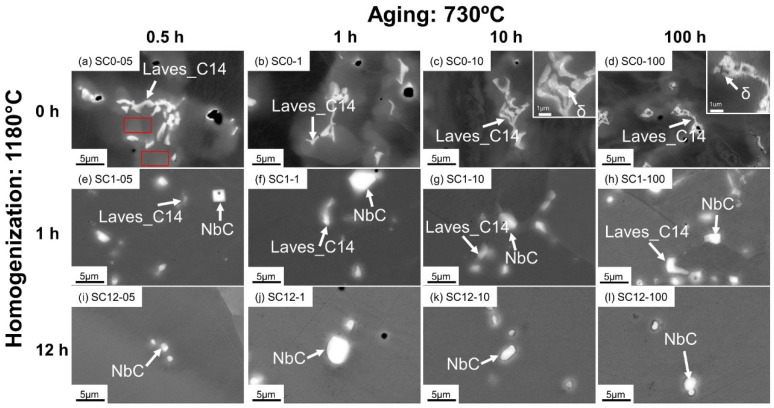
SEM-BSE micrographs of the direct-aged SC samples ((**a**) SC0-05; (**b**) SC0-1; (**c**) SC0-10; (**d**) SC0-100), the aged SC samples homogenized at 1180 °C for 1 h ((**e**) SC1-05; (**f**) SC1-1; (**g**) SC1-10; (**h**) SC1-100), and the aged SC samples homogenized at 1180 °C for 12 h ((**i**) SC12-05; (**j**) SC12-1; (**k**) SC12-10; (**l**) SC12-100).

To evaluate the manufacturing impact on microstructure evolution and obtain more insight into the phase transformations to better guide heat treatment optimization, microstructure characterization was performed on the SC alloys with the same heat treatment conditions as the AM alloys. From Figure 4a–d, the direct-aged SC samples retain a microstructure identical to that of the as-cast sample, as reported in [5], and the irregular Laves_C14 phase precipitates in the γ matrix. Compared with the Laves_C14 particles in the AM alloys, e.g., in Figure 2a, the Laves_C14 particles in the SC alloys are larger in size, and are enclosed by a visible Nb-rich microsegregation region, which is indicated by the red rectangles with a brighter gradient contrast from the Laves phase to the γ matrix under the backscattered electron signal in Figure 4a. This phenomenon was also reported in the work of Rafiei et al. [25]. It is due to the higher segregation generated during solidification in the suction casting process, which has a much slower cooling rate than the PBF-LB/M process [26,27,28]. After 10 h aging, the δ phase starts forming at the phase boundary between the Laves_C14 and γ, as shown in the inset of Figure 4c. After 100 h aging, the δ particles continue to grow into the needle shape, and are easier to observe, as demonstrated in Figure 4d and its inset, due to the same reason as discussed for sample AM0-100.

In the aged samples after 1 h homogenization (Figure 4e–h), NbC carbides were found. However, because the homogenization process is incomplete, some Laves_C14 particles remain undissolved in the homogenized samples, and coexist with the NbC carbides during the following aging process. In contrast to the direct-aged samples, δ precipitates can hardly be identified in the aged samples after 1 h homogenization. With 12 h homogenization at 1180 °C, the Laves_C14 phase is completely dissolved, and NbC carbides are observed to be predominant in the SEM images of the as-polished samples.

**Figure 5:**
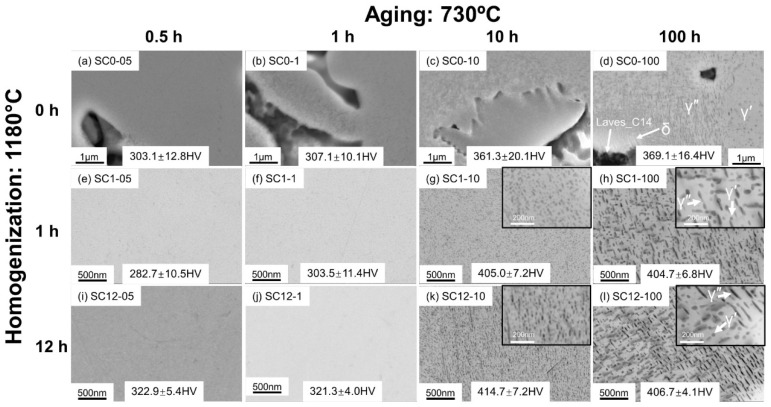
SEM-SE micrographs of the direct-aged SC samples after etching ((**a**) SC0-05; (**b**) SC0-1; (**c**) SC0-10; (**d**) SC0-100), the aged SC samples homogenized at 1180 °C for 1 h after etching ((**e**) SC1-05; (**f**) SC1-1; (**g**) SC1-10; (**h**) SC1-100), and the aged SC samples homogenized at 1180 °C for 12 h after etching ((**i**) SC12-05; (**j**) SC12-1; (**k**) SC12-10; (**l**) SC12-100).

The precipitation traces of γ″ and γ′ in the direct-aged samples become more obvious after 10 h aging (sample SC0-10). According to Figure 5c,d, such precipitates are not distributed homogeneously during aging. After 100 h aging, as shown in Figure 5d, the γ matrix is divided into various regions that are dominated by different precipitates. The δ phase precipitates on the phase boundaries attaching to the Laves_C14 phase, whereas γ″ particles mainly precipitate in a farther region. The γ′ precipitates dominate the region farthest from the Laves_C14 particles, indicating an Nb-depletion but an Al and Ti-rich area. The precipitation behaviors of the unevenly distributed γ″ and γ′ in the direct-aged SC samples can be explained similarly due to the Nb segregation, as discussed in Section 3.1 for the AM alloys. Such an observation strongly indicates that the inherited heterogeneity introduced during the manufacturing process cannot be easily eliminated during aging without homogenization treatment. However, the heterogeneous region is much larger in the SC samples than in the samples prepared by PBF-LB/M due to the higher segregation degree caused by the slower solidification rate. A similar phenomenon has been reported by Rafiei et al. [25], who used EDS line scanning to confirm the gradual change in Nb content near the Laves_C14 particles, and concluded that the Nb-bearing phases tend to precipitate in the regions with higher Nb content.

As shown in Figure 5e–h, in the aged samples homogenized for 1 h at 1180 °C, the γ″ and γ′ phases start to precipitate after 10 h aging, and sufficiently grow with 100 h aging. The distribution of γ″/γ′ precipitates is moderately homogeneous, which is in contrast to the case in the direct-aged samples, as shown in Figure 5d. A similar phase transformation behavior was found in the samples homogenized for 12 h, as can be seen in Figure 5i–l, with a starting formation time of 10 h for the γ″/γ′, and an even distribution of these precipitates after 100 h aging. Further quantitative analysis revealed the difference in the precipitation attributes between the 100 h aged SC samples with different homogenization conditions. Moreover, an intuitive comparison of the precipitation attributes between the AM and SC alloys after 100 h aging, as demonstrated in Figure 3h,l and Figure 5h,l, implies that the precipitates in the AM alloys exhibit smaller particle sizes and a higher number density than their counterparts in the SC alloys. A specific discussion on the variety of precipitation attributes will be given in Section 3.3.

### 3.3. Precipitation Attributes Affected by Homogenization and Manufacturing

The precipitation attributes of γ″ and γ′, such as particle size and number density, were quantitatively analyzed by image analysis. This will help us to understand the effects of homogenization time and manufacturing methods on the phase transformations during the aging process. Since long-term aged samples are important for the engineering application of Inconel 718, samples AM1-100, AM12-100, SC1-100, and SC12-100 were analyzed. Moreover, the precipitate growth in these samples is sufficient, and thus allows more accurate quantification. At least 1180 particles were analyzed for each sample to ensure the results were representative. The results of the precipitation attribute analysis are summarized in Table 3.

It was found that the longer homogenization time in the aged AM samples led to an increase in the particle size for both γ″ and γ′ phases, while the number density of the strengthening particles was reduced. Nevertheless, a reversed tendency was found in the SC alloys, among which the particle size of the γ″ and γ′ precipitates in the aged samples with long-term homogenization was smaller, yet the number density was higher. Because aging treatments are applied in the same way to both AM and SC alloys, it can be speculated that the microstructure obtained after the homogenization heat treatment plays an essential role in microstructure evolution, including precipitation kinetics. Additionally, it has been known that the Nb content distribution can affect the precipitation behaviors of γ″ and γ′ particles, as discussed in Section 3.1 and Section 3.2. Therefore, understanding the influence of homogenization on Nb distribution before aging becomes critical.

**Figure 6:**
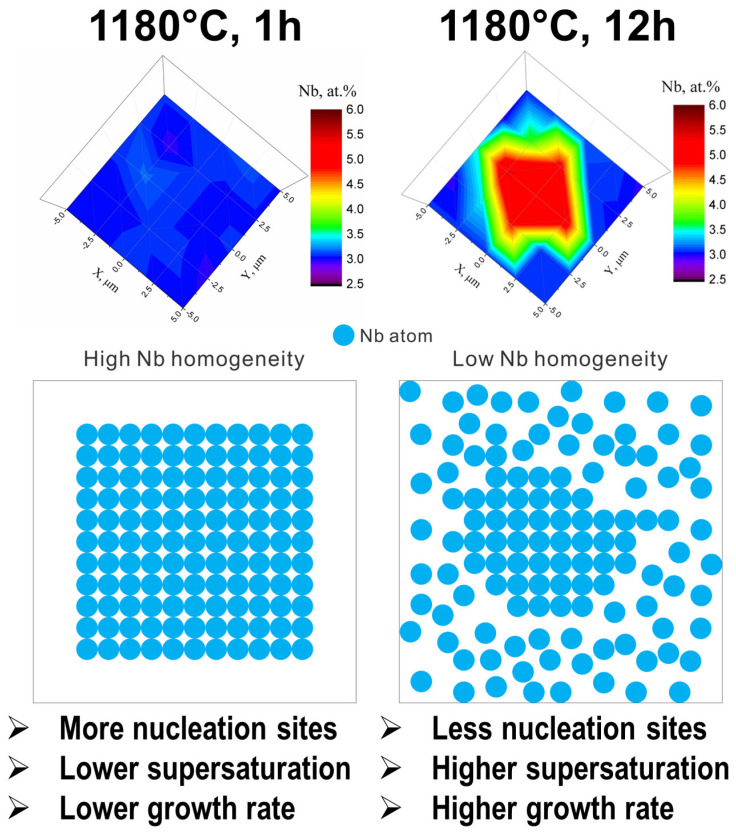
Illustration of the impacts of Nb homogeneity on the precipitation attributes of γ″/γ′ in the AM alloys.

Zhao et al. [5] reported that in the alloys produced by PBF-LB/M, 12 h homogenization leads to a decrease in the Nb homogeneity compared with the 1 h homogenization case, yet an opposite situation occurs in the SC alloys. Based on the conclusions made in [5], the precipitation attributes affected by the homogenization time in both alloys can be tentatively explained. For instance, for the AM alloys, as shown in Figure 6, the Nb homogeneity in the γ matrix after 1 h homogenization is higher, resulting in every Nb atom sharing the same probability of becoming a potential nucleation site for γ″/γ′. Therefore, the nucleation sites are increased, which, accordingly, raises the number density of the precipitates in sample AM1-100, as indicated in Table 3. Additionally, the high Nb homogeneity reduces due to the supersaturation of Nb content in the matrix, which decreases the precipitate growth rate [29]. Papadaki et al. [21,30] also indicated that the local concentration gradients and elemental redistribution impact the precipitation behaviors in Ni-based superalloys, which is similar to this study. Consequently, the particle sizes of γ″ and γ′ are smaller in sample AM1-100 (Table 3). The same analysis can be applied to interpret the effects of the homogenization time on the precipitation attribute evolution in the SC alloys.

Moreover, the comparisons of precipitation attributes between samples AM1-100 and SC1-100, as well as between samples AM12-100 and SC12-100, indicate that, under the same heat treatment condition, the AM samples generate smaller particle sizes and a higher number density than the corresponding SC samples. The Nb homogeneity difference between AM1-100 (high Nb homogeneity with more nucleation sites and lower growth rate) and SC1-100 (low Nb homogeneity with fewer nucleation sites and higher growth rate) can explain the change in precipitation attributes in these two samples based on the mechanism proposed above. However, it fails to explain the difference between the precipitation attributes of AM12-100 and SC12-100. This may be because the dissolution of the Laves phase in SC alloys during 12 h homogenization introduced more dislocations, which can be heterogenous nucleation sites and diffusion pipes. Therefore, the number density of particles is reduced, but the particle size increased due to the higher diffusion kinetics. Despite the proposed tentative explanation, the difference in precipitation attributes between the AM and SC alloys with the same heat treatment should be further investigated, especially through some mechanistic modeling, in the future.

### 3.4. Microhardness

**Figure 7:**
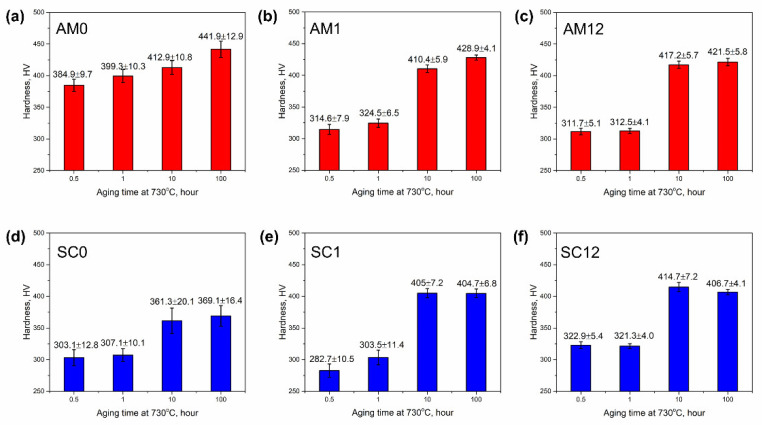
Microhardness of aged samples: (**a**) AM alloys without homogenization; (**b**) AM alloys with 1 h homogenization; (**c**) AM alloys with 12 h homogenization; (**d**) suction-cast alloys without homogenization; (**e**) suction-cast alloys with 1 h homogenization; (**f**) suction-cast alloys with 12 h homogenization.

The Vickers microhardness values of the aged samples are summarized in Figure 7. For each alloy with the same manufacturing and homogenization conditions in Figure 7, the hardness values increase significantly after 10 and 100 h aging. However, in the AM alloys without homogenization (group AM0), the samples AM0-05 and AM0-1 have higher hardness values than all other samples aged for 0.5 or 1 h, regardless of the manufacturing and homogenization conditions. This can be attributed to the retained fine subgrains in the AM0 alloys that increase the hardness, as shown in Figure 2a,b. Compared with the samples of group AM0, the aged SC samples without homogenization (group SC0) have a lower hardness than their counterparts with the same aging conditions.

**Figure 8:**
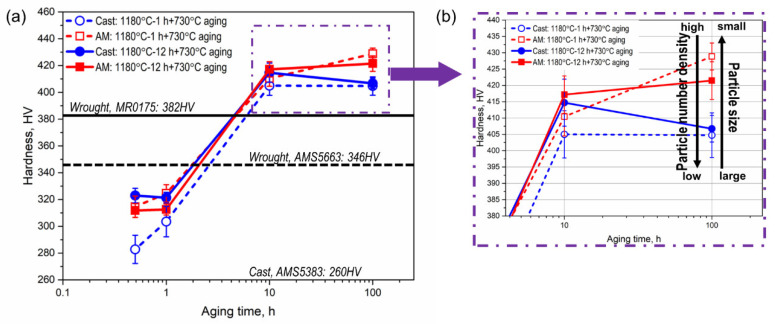
Microhardness evolution in the aged AM and SC alloys after homogenization: (**a**) the microhardness evolution from 0.5 to 100 h aged samples; (**b**) magnified figure for the microhardness evolution of 10 and 100 h aged samples.

The Vickers microhardness of the aged samples after homogenization is illustrated in Figure 8 to further elucidate the effect of homogenization time and manufacturing on hardness evolution. It is obvious from Figure 8a that the microhardness values are comparable between the 0.5 and 1 h aged samples. However, the microhardness exhibits a jump from 1 to 10 h aging in all the groups of samples, and the hardness values of the 10 h aged samples are superior to that of the commercial wrought alloys, as shown in Figure 8a. The increase in hardness after 10 h aging is due to the precipitation hardening stems from the γ″/γ′ strengthening phases formed after 10 h, which conforms with the microstructure observation in Figure 3e–l and Figure 5e–l. From Figure 8b, it can be seen that with the 100 h aging that allows the precipitates to grow sufficiently, the hardness values of the AM samples are higher than those of the SC samples. The superior hardness achieved in the AM samples is owing to the relatively smaller precipitate size and the higher number density obtained compared with the counterparts in the SC samples, as shown in Table 3 and discussed in Section 3.3. The small particle size indicates that the interface between the strengthening particles and the γ matrix is higher in coherency, and promotes the hardening effects in Inconel 718 [31,32,33].

### 3.5. Grain Morphology

**Figure 9:**
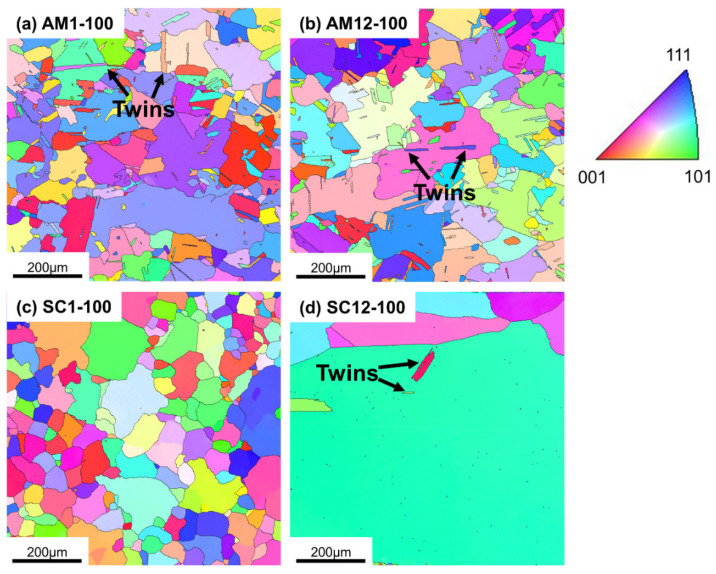
Grain morphologies of 100 h aged samples after homogenization by EBSD: (**a**) sample AM1-100; (**b**) sample AM12-100; (**c**) sample SC1-100; (**d**) sample SC12-100.

The grain morphologies of the 100 h aged samples after homogenization were determined by EBSD mapping, which is shown in Figure 9. As can be seen in Figure 9a,b, the grain morphology in the AM samples is equiaxial, and annealing twins formed during homogenization [5,34,35] can be easily identified. The twins are beneficial for the strength and ductility of the alloy [36,37]. In the aged SC samples indicated by Figure 9c,d, the grain morphologies are equiaxial, with the twins found in sample SC12-100, whereas the amount is less than that in the AM samples. The annealing twins were rarely found in the 1 h homogenized SC sample, and they could only be tracked in the 12 h homogenized sample [5]. The results indicate that the twins in the SC samples mainly form during the homogenization process, and their formation needs a longer homogenization time than that in the AM samples, which only takes less than 1 h.

**Figure 10:**
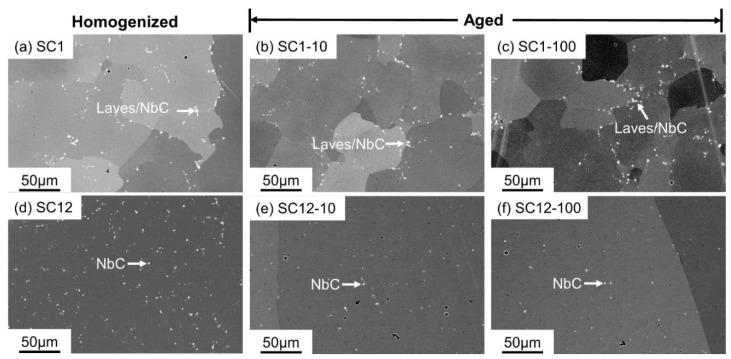
SEM-BSE micrographs of the SC alloys: (**a**) sample SC1, which is homogenized at 1180 °C for 1 h; (**b**) sample SC1-10; (**c**) sample SC1-100; (**d**) sample SC12, which is homogenized at 1180 °C for 1 h; (**e**) sample SC12-10; (**f**) sample S12-100.

Table 4 summarizes the average grain sizes of the aged samples and the corresponding homogenized samples, which were obtained from EBSD characterization. The grain size of sample SC12-100 is too large to be counted in this work, which has a negligible effect on the conclusions. As indicated by Table 4, the grain sizes of samples AM1-100 and AM12-100 are comparable with their corresponding homogenized counterparts [5], implying that the aging process at 730 °C cannot promote grain growth in PBF-LB/M alloys remarkably. The sample SC12-100 has a significantly coarsened grain size due to abnormal grain growth during homogenization [5]. Interestingly, notable grain refinement can be found in sample SC1-100 (Table 4 & Figure 9c), of which the grain size is 100.9 ± 54.9 μm, which is much smaller than that of the corresponding homogenized sample. Further microstructure characterization was performed on the aged SC samples after 1 h homogenization, and the corresponding aged samples after 12 h homogenization are also shown as a comparison. As shown in Figure 10b,c, the Laves_C14 or NbC particles are distributed along the grain boundaries, which may impede the grain growth due to the Zener pinning effect. In contrast, most of the NbC particles reside in the interior of the grains in samples SC12-10 and SC12-100, and cannot act as the pinning particles, as illustrated in Figure 10e,f. In fact, although the impediment of the grain growth in sample SC1-100 can be explained by the Zener pinning effects, the mechanism of the grain refinement requires more comprehensive studies. One possible account for the grain refinement might be the introduction of dislocations by the dissolution of the Laves_C14 phase during aging; the dislocations can glide and tangle to form subgrain boundaries [38,39], which further form the high-angle grain boundaries [38]. However, a further specific study is needed to reveal the mechanism.

## 4. Concluding Remarks

1. Both homogenization and manufacturing conditions exert a significant influence on the precipitation kinetics, affecting the alloy’s strengthening process. By adopting homogenization, the precipitation homogeneity of strengthening phases in both aged AM and suction-cast alloys can be improved. At 730 °C, the precipitation of γ″ and γ′ strengthening phases becomes more pronounced after 10 h aging, irrespective of homogenization and manufacturing conditions. Consequently, customized optimization and design of post-heat treatment strategies are essential for AM alloys.
2. The homogenization time has a direct impact on the precipitation attributes of γ″ and γ′ in adequately aged Inconel 718. An extended homogenization time in AM alloys results in increased particle size but decreased precipitate number density, while the trend is reversed in suction-cast alloys. The disparity in Nb homogeneity arising from alloy homogenization can account for the observed variations in precipitation attributes.
3. The choice of manufacturing method influences the precipitation attributes of γ″ and γ′. The AM alloys exhibit smaller particle sizes but higher number densities compared to suction-cast alloys with the same heat treatment.
4. Microhardness values of aged Inconel 718 demonstrate a significant increase after 10 h aging, irrespective of homogenization and manufacturing conditions, primarily due to the precipitation hardening effect of γ″ and γ′. After 100 h aging, the AM alloys exhibit higher hardness than suction-cast alloys with the same heat treatment, owing to the finer strengthening particle size and higher number density.
5. Overall, the grain size of aged Inconel 718 is generally comparable to that of the corresponding homogenized alloys. However, specific heat treatment and manufacturing conditions can induce grain refinement. The complete underlying mechanism of this phenomenon remains elusive, necessitating further detailed investigations into this intriguing topic.

## Figures and Tables

**Table 1:** Nominal compositions of alloying elements in AM and SC alloys.

wt.%	Ni	Fe	Cr	Nb	Mo	Ti	Al
SC	Bal.	18.50	18.30	4.99	3.04	1.02	0.55
AM	Bal.	18.26	18.87	4.97	2.97	0.94	0.46
**wt.%**	**Mn**	**Co**	**Cu**	**Si**	**C**		
SC	0.23	0.39	0.07	0.08	0.051		
AM	0.06	0.23	0.05	0.06	0.03		

**Table 2:** Sample notations and corresponding heat treatments for different alloys.

AM-PBF-LB/M			Suction Casting		
Sample	Homogenization	Aging time at 730 °C, hour	Sample	Homogenization	Aging time at 730 °C, hour
AM0-05	As-built	0.5	SC0-05	As-cast	0.5
AM0-1		1	SC0-1		1
AM0-10		10	SC0-10		10
AM0-100		100	SC0-100		100
AM1-05	1180 °C for 1 h	0.5	SC1-05	1180 °C for 1 h	0.5
AM1-1		1	SC1-1		1
AM1-10		10	SC1-10		10
AM1-100		100	SC1-100		100
AM12-05	1180 °C for 12 h	0.5	SC12-05	1180 °C for 12 h	0.5
AM12-1		1	SC12-1		1
AM12-10		10	SC12-10		10
AM12-100		100	SC12-100		100

**Table 3:** Precipitation attributes of γ″ and γ′ in 100 h aged samples after homogenization.

Sample	γ″ Particle Length, nm	γ′ Particle Length, nm	Number Density (γ″ + γ′), /m^2^
AM1-100	45.39 ± 26.87	21.98 ± 11.06	2.8 ± 0.29 × 10^14^
AM12-100	51.21 ± 30.83	23.95 ± 11.68	2.21 ± 0.84 × 10^14^
SC12-100	70.32 ± 42.4	34.87 ± 17.57	9.65 ± 0.41 × 10^13^
SC1-100	86.68 ± 46.83	35.48 ± 16.26	9.03 ± 0.86 × 10^13^

**Table 4:** The average grain size of the 100 h aged samples after homogenization and the corresponding homogenized samples.

Average Grain Size, μm	AM1-100	AM12-100	SC1-100	SC12-100
Aged sample	113.4 ± 74.5	115.7 ± 62.7	100.9 ± 54.9	-
Homogenized sample *	128.8 ± 75.2	113.6 ± 63.6	302.9 ± 128.7	648.2 ± 292.7

* The grain size values of the homogenized samples are reported in reference [5].

## Data Availability

All relevant data are contained in the present manuscript.

## Supplementary Materials

The following supporting information can be downloaded at: https://www.mdpi.com/article/10.3390/ma16144968/s1.
