# Supplementary material for: Influence of Homogenization on Phase Transformations during Isothermal Aging of Inconel 718 Superalloys Fabricated by Additive Manufacturing and Suction Casting
Source: Materials (Basel). 2023 Jul 12;16(14):4968. doi: 10.3390/ma16144968 (PMC10383715; doi:10.3390/ma16144968)
Supplement: Supplementary file 1 [file materials-16-04968-s001.zip › materials-2437961-supplementary.pdf]

Supplemental Information

1. Printing parameters for PBF-LB/IN718 builds in this research

Inert Shielding Atmosphere: Argon Gas

Layer Thickness: 40 Micron

Infill:

Exposure Pattern: Stripes

Stripe Width:10mm

Laser Power: 285W

Laser Speed: 960mm/s

Hatching Distance: 0.11mm

Hatch Rotation: 67 Degrees

Upskin:

No Exposure Pattern

3 Layers Thick

Double Exposure Enabled

Laser Power 153W

Laser Speed 600mm/s

Hatch Distance 0.09mm

Contour Set #1

Offset:0.012mm

Corridor:0.04mm

Std. Contour:138W, 300mm/s

Contour Set #2

Offset: 0mm

Corridor:0.04mm

Std. Contour:80W, 800mm/s

Edge

Offset:0mm

Edge Factor:2

Threshold:3

Min. Radius Factor:0

Laser Power:100W

Laser Speed 900mm/s
